# Case Report: Trissing balloon inflation and percutaneous coronary intervention with drug-coated balloons for the treatment of restenosis of a left main trifurcation lesion

**DOI:** 10.3389/fcvm.2025.1558491

**Published:** 2025-05-12

**Authors:** Francesco Amata, Francesco Paolo Gioia, Gaetano Liccardo, Giancarlo Barberis, Giuseppe Ferrante

**Affiliations:** ^1^ Department of Cardiovascular Medicine, IRCCS Humanitas Research Hospital, Milan, Italy; ^2^ Department of Biomedical Sciences, Humanitas University, Milan, Italy

**Keywords:** left main trifurcation, left main restenosis, left main angioplasty, in-stent restenosis, complex PCI

## Abstract

We report the case of a 62-year-old male with multiple cardiovascular risk factors and comorbidities who presented to our institution due to unstable angina. One year earlier, he underwent percutaneous coronary intervention (PCI) to unprotected left main trifurcation lesion involving the ostial left anterior descending artery (LAD) (Medina classification 0-0-1-0) with provisional stenting technique with single drug-eluting stent (DES) implantation from left main to LAD and PCI to LAD with single DES implantation from LAD in crossover with D1 for the treatment of LAD-D1 bifurcation lesion (Medina 1-1-0). Coronary angiography by radial approach found sub-occlusive restenosis of both jailed ostial ramus intermediate (RI) and left circumflex (LCX), with patency of DES to left main LAD and a significant in-stent restenosis (ISR) of DES to LAD at the bifurcation with D1. LAD ISR was treated with PCI with single DES implantation with optimal angiographic results. The left main trifurcation restenosis was treated by radial approach PCI with simultaneous trissing balloon inflation to left main, RI, and LCX, followed by kissing balloon with drug-coated balloons with sirolimus elution to RI and LCX, subsequent trissing balloon inflation, and final proximal optimization technique to the left main achieving an optimal angiographic result. Planned follow-up angiography at 1 year showed persistence of optimal angiographic results.

## Introduction

The left main coronary artery can divide into three branches (trifurcation), i.e., left anterior descending artery (LAD), left circumflex (LCX), and an additional branch, usually called ramus intermediate (RI) or anterolateral branch, in 6.7% to 52.2% of cases (1–4). High variability of left main branching patterns has been found across different ethnic groups, sexes, and different body surface areas (1), with cases of quadri- or penta-furcations also (5, 6). Left main trifurcations have two carinas (between LAD and RI and between RI and LCX) and four angles among the three branches (1). This anatomical configuration is associated with high flow turbulence and shear stress abnormalities leading to atherosclerotic plaque formation with heterogenous distribution, size, and characteristics across the different branches (1–7).

Left main trifurcation lesions can be classified using a modified Medina classification in the form of a four-digit number (e.g., 1-0-1-1 or 1-0-1-1-1) indicating significant involvement of the main branch and both side branches (8). Another classification uses a letter-based nomenclature system to indicate the diseased vessel (e.g., A, LM; B, LAD; C, LCX; and D, RI), with capital letters indicating vessels ≥3.5 mm and lowercase letters for vessel <3.5 mm (9). A stenosis is considered significant if it is >70% on angiography, according to intravascular imaging criteria, or based on functional evaluation (9).

Moreover, the complexity of anatomic configurations of left main trifurcation lesions poses challenges for interventional cardiologists owing to the risk of procedural complications, such as carina or plaque shift and side branch occlusion, and the need for more complex procedures in some cases (10, 11). Furthermore, challenges may arise when restenosis occurs following successful percutaneous coronary intervention (PCI) of left main trifurcation lesions.

We report a case of left main trifurcation restenosis successfully treated with a metal-free percutaneous coronary intervention (PCI).

## Case presentation

**Figure 1:**
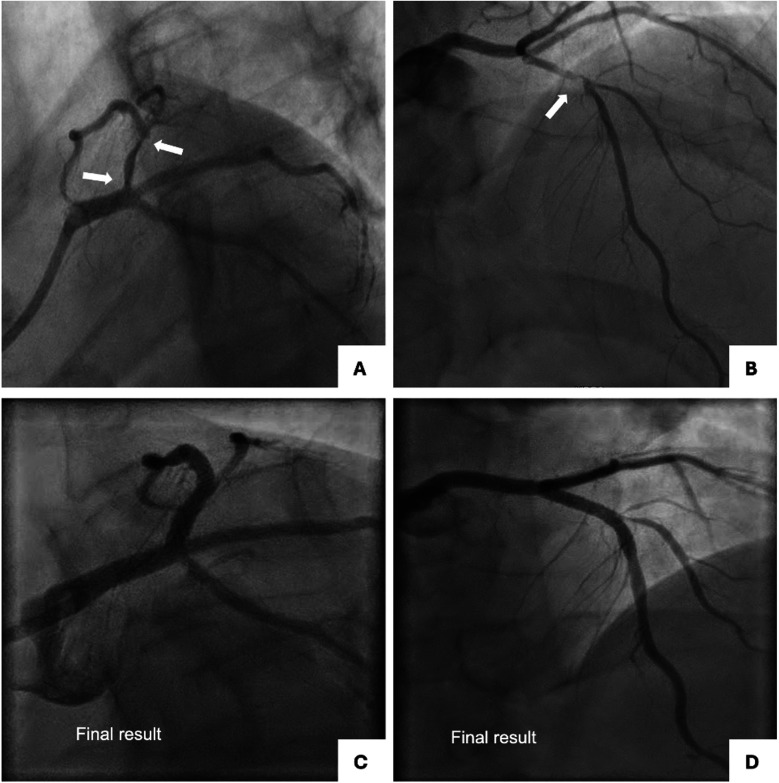
Baseline and final coronary angiography at the index procedure. **(A)** Spider view showing trifurcated LM coronary artery with severe ostial and proximal LAD (modified Medina 0-0-1-0; A, LM; B, LAD). **(B)** Right oblique cranial view showing severe LAD disease at the level of the bifurcation with D1 (Medina 1-1-1). **(C)** Spider view showing final result after provisional single DES stenting from LM to LAD. **(D)** Right oblique cranial view showing the final result after provisional single DES stenting to LAD in crossover. Pinching of jailed ostial D1 with TIMI 3 flow. D1, first diagonal branch; DES, drug-eluting stent; LAD, left anterior descending artery; LM, left main.

**Figure 2:**
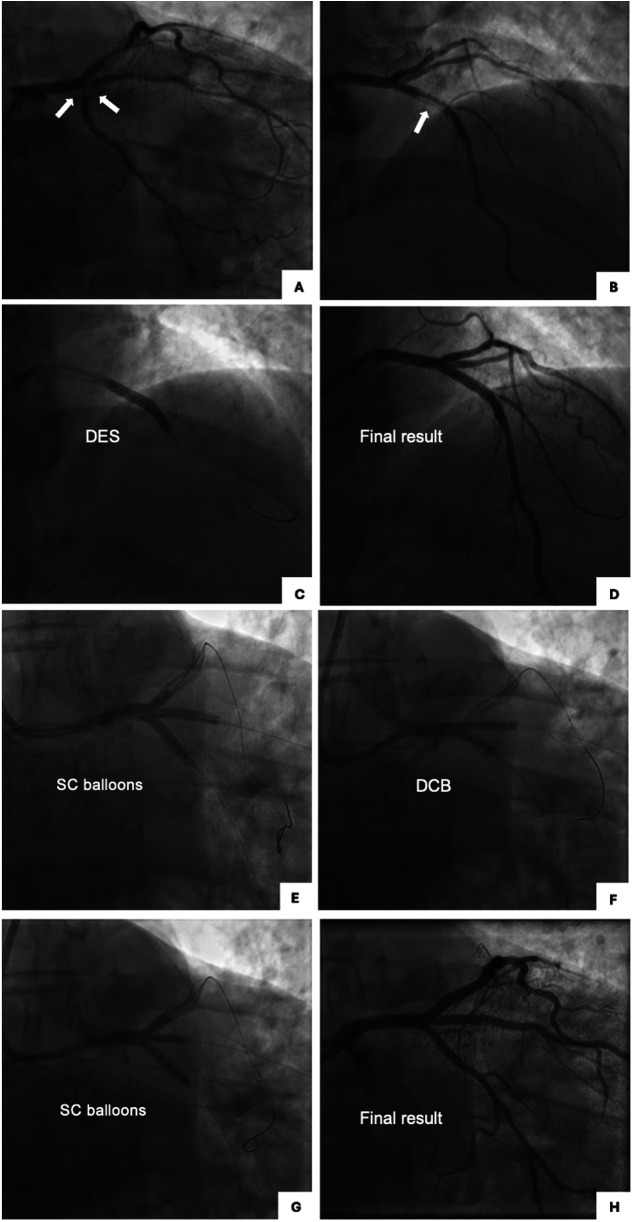
One-year follow-up coronary angiography: baseline, steps of the procedure, and final result. **(A)** Right oblique caudal view showing patency of DES to LM-LAD with severe stenosis of jailed ostial RI and LCX. **(B)** Right oblique cranial view showing significant ISR of DES to LAD, unchanged pinching of jailed ostial D1 with TIMI 3 flow. **(C,D)** Right oblique cranial view showing DES implantation to LAD, after predilation with SC 3.0 × 15 mm and NC 3.5 × 15 mm balloons for the treatment of ISR **(C)**. Final result after DES implantation **(D)**. **(E–G)** Right caudal view showing treatment of jailed ostial RI and LCX: trissing balloon using 3.0 × 15 mm SC balloons **(E)**, kissing balloon to LM-RI and LM-LCX with DCB **(F)**, trissing balloon with 3.0 × 15 mm SC balloons **(G)**. **(H)** Right caudal view showing final result after POT with NC 4.5 mm balloon. D1, first diagonal branch; DCB, drug-coated balloon; DES, drug-eluting stent; ISR, in-stent restenosis; LAD, left anterior descending artery; LCX, left circumflex artery; LM, left main; NC, non-compliant; RI, ramus intermediate; SC, semi-compliant.

**Figure 3:**
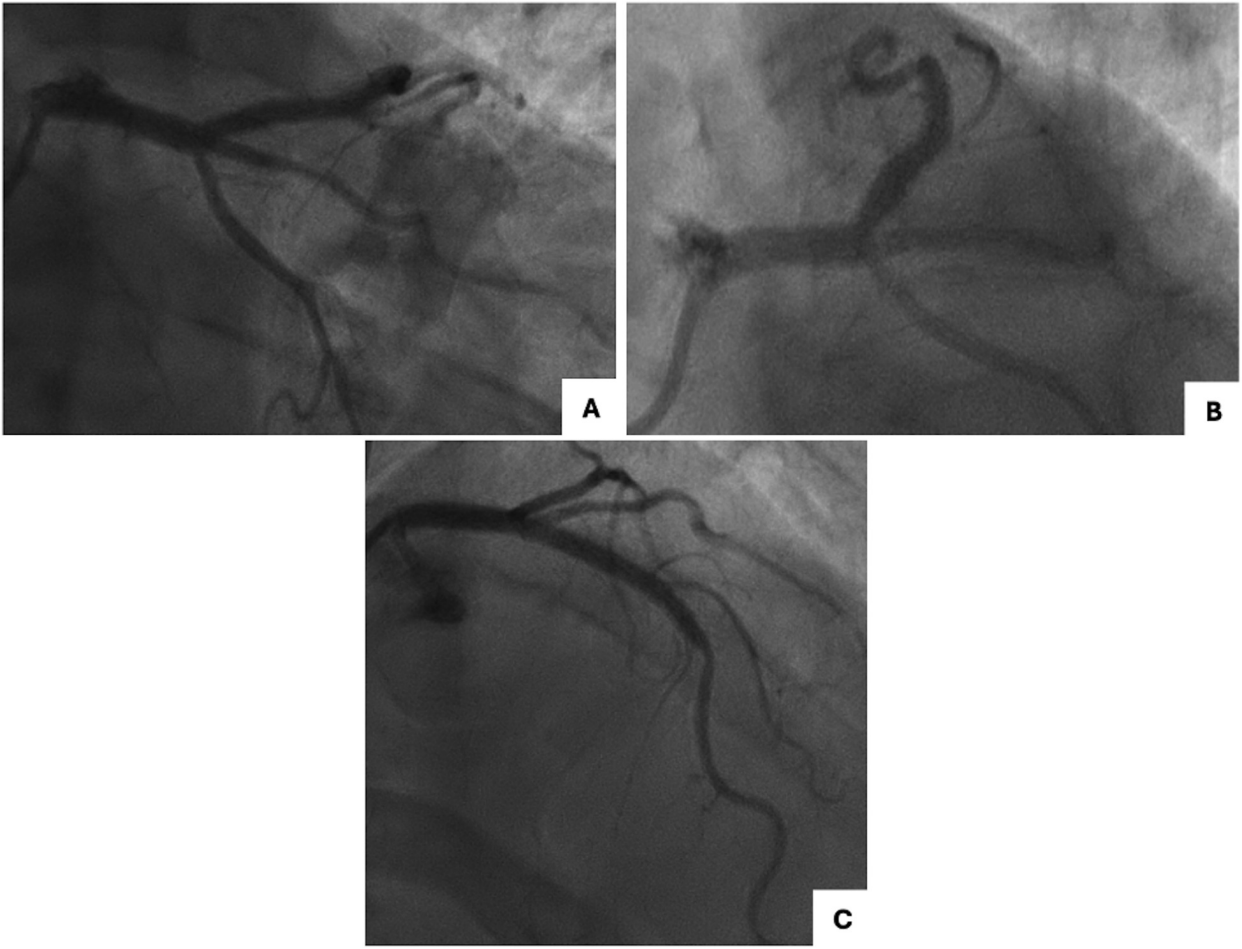
One-year follow-up coronary angiography following treatment of ISR. **(A,B)** Right oblique caudal view **(A)** and spider view **(B)** showing optimal result of DCB at the ostium of jailed RI and LCX and patency of DES to LM-LAD. **(C)** Right oblique cranial view showing patency of DES to LAD, unchanged pinching of jailed ostial D1 with TIMI 3 flow. D1, first diagonal branch; DCB, drug-coated balloon; DES, drug-eluting stent; LAD, left anterior descending artery; LCX, left circumflex artery; LM, left main.

A 62-year-old male, former smoker, with diabetes mellitus, family history of coronary artery disease, hypertension, dyslipidemia, prior orthotopic liver transplant due to HCV-related cirrhosis, and chronic thrombocytopenia and leukopenia attributed to hypersplenism and antirejection immunosuppressive drugs, underwent intravascular ultrasound (IVUS)-guided PCI to unprotected left main trifurcation lesion involving ostial LAD (Medina classification 0-0-1-0) with provisional stenting technique with single drug-eluting stent (DES) implantation from left main to LAD and PCI to LAD with single DES implantation, in crossover with D1 and overlapping stenting, for the treatment of LAD-first diagonal (D1) bifurcation lesion (Medina 1-1-0) (Figures 1A,B). A good angiographic result was achieved with minor angiographic stenosis of jailed LCX and pinching of jailed ostial D1 with TIMI 3 flow (Figures 1C,D). Clinical follow-up was uneventful until 1 year after the index procedure, when the patient started complaining of new-onset effort angina (Canadian Cardiovascular Society Class II) that rapidly progressed to unstable angina, leading to admission to our institution. Coronary angiography by radial approach found sub-occlusive restenosis of both jailed ostial RI and LCX with patency of the DES to left main LAD and a significant in-stent restenosis (ISR) of the DES to LAD at the LAD-D1 bifurcation lesion with unchanged pinching of the jailed D1 (Figures 2A,B). Blood tests showed stable and unchanged thrombocytopenia (50,000 mm^3^) and leukopenia (3,500 mm^3^). After the patient refused coronary artery bypass graft, revascularization was performed by PCI by a radial approach using a 7-French guiding catheter through a 7-French thin-wall introducer. LAD ISR was treated with PCI with DES implantation (3.5 × 22 mm), following lesion preparation with a non-compliant 3.5 × 15 mm balloon, achieving a good angiographic result with unchanged pinching of jailed ostial D1 with TIMI 3 flow (Figures 2C,D). The left main trifurcation restenosis was treated with simultaneous trissing balloon inflation using 3.0 × 15 mm semi-compliant balloons to left main LAD-RI-LCX (Figure 2E), followed by kissing balloon dilation with 3.0 × 20 mm DCBs with sirolimus release to left main RI and left main LCx (Figure 2F), subsequent trissing balloon dilation with 3.0 × 15 mm semi-compliant balloons (Figure 2G), and final proximal optimization technique (POT) with 4.5 mm non-compliant balloon to left main achieving an optimal angiographic result (Figure 2H). The patient was discharged home with the indication to continue dual antiplatelet therapy with aspirin and clopidogrel for 6 months followed by aspirin monotherapy. No change in platelet count throughout the follow-up was observed. At the 1-year follow-up, the patient remained asymptomatic and underwent a planned coronary angiography which showed patency of DES to left main LAD without restenosis, patency of jailed RI and LCX with no restenosis, and patency of the DES to LAD bifurcation lesion with unchanged pinching of jailed ostial D1 with TIMI 3 flow (Figure 3).

## Discussion

PCI with DES is recommended as an alternative therapeutic option to coronary artery bypass graft surgery for the treatment of unprotected left main coronary artery disease in patients with low SYNTAX score and should be considered in patients with intermediate SYNTAX score (12, 13). In addition, PCI with DES to left main may also be considered in patients with multiple comorbidities and very high surgical risk or case of patient refusal for CABG.

Nevertheless, there is limited evidence on the optimal revascularization strategy with PCI for left main trifurcation lesions. Data from the EXCEL trial showed no significant differences between left main trifurcation and left main bifurcation lesions with respect to major adverse cardiovascular events (MACE) at 30 days and 5-year follow-up (14).

In our case, the left main trifurcation lesion at the time of the index procedure was a Medina 0-0-1-0 or B. Both the left main trifurcation and the LAD-D1 bifurcation lesions were treated with a simple approach consisting of a provisional single-stent strategy from the main vessel across the side branch. The decision to use a provisional one-stent strategy with wiring protection of side branches vs. two- or three-stent strategies should consider anatomical factors such as plaque burden distribution across the trifurcation, the presence of calcification or calcified nodules, the risk of acute side branch occlusion, and technical difficulty in recrossing very angulated side branches across stent struts after main vessel crossover stenting. Two- or three-stent strategies should be used for anticipated very complex lesions with a high risk of side branch occlusion after main vessel crossover stenting and as a bailout for flow-limiting dissections of side branches (8, 9).

The selection of the optimal treatment options for ISR following left main trifurcation-PCI remains largely undefined. The initial stenting strategy may affect the type of ISR and its subsequent management: e.g., provisional single stenting from left main to LAD vs. two- or three-stent techniques. Intravascular imaging guidance with IVUS and/or optical coherence tomography (OCT) is useful for left main PCI (15, 16) as well as for understanding the mechanisms of restenosis (17, 18). Indeed, intravascular imaging allows to diagnose stent under-expansion, strut malapposition, deformation of neocarina geometry, such as excessive protrusion of side branch stent struts into the main vessel (TAP technique), incomplete side branch ostium coverage (T stenting), ring-like pattern of restenosis at the carina (culotte technique), and protruding calcified nodules across stent struts (17, 18). OCT also allows the characterization of the pattern in-stent restenosis distinguishing between neointimal hyperplasia and neo-atherosclerosis as well as between aggressive ISR and stent thrombosis in cases of sub-occlusive lesions with uncertain angiographic appearance (17, 18).

The use of a metal-free PCI strategy with drug-coated balloon (DCB) for the treatment of left main trifurcation restenosis is desirable to reduce stent overlapping and minimize the risk of subsequent target lesion failure, in particular in patients treated with two- or three-stent techniques at the index procedure, such as DK-crush, TAP, or culotte stenting (19).

Evidence for the safety and efficacy of treatment of *de novo* lesions with a DCB-only strategy is rapidly growing (20, 21) and the role of DCB in the treatment of bifurcation lesions is emerging (22–25). A few studies have combined a strategy of PCI with DES to the main vessel and DCB to the side branch, reporting good angiographic results with low rates of target lesion revascularization and restenosis at follow-up (26–29). The value of PCI with DCB as compared to PCI with DES for the treatment of ISR following DES has also been shown (30). Adequate lesion preparation before DCB use is important for achieving an effective result (31). Optimal ostial side branch expansion with balloon- or non-balloon-based techniques, according to intravascular imaging, should be performed. For example, semi- or non-compliant balloons may be sufficient to expand side branch ostia across stent struts in the main vessel in most cases. However, additional techniques including cutting balloons, scoring balloons, very high-pressure NC balloons, and/or intravascular lithotripsy may be used according to intravascular imaging findings (32, 33). Nevertheless, data about the use of DCB for the treatment of restenosis of left main trifurcation lesions are lacking.

Potential advantages of DCB use for the treatment of left main ISR and restenosis of jailed side branches in complex anatomy, such as trifurcated left main, include the avoidance of additional stent implantation and strut overlap, as well as neocarina formation with imperfect strut orientation which may increase the risk of ISR and stent thrombosis (25, 34). In addition, the use of a free-metal PCI strategy may allow a safe reduction of dual antiplatelet therapy duration, compared to double stenting techniques, which may translate into an additional clinical benefit in patients at high-bleeding risk. Furthermore, DCBs may promote positive vessel remodeling at follow-up (35, 36). Nevertheless, DCB use, compared with DES implantation, may be associated with smaller acute minimal lumen areas and carries a higher risk of acute dissections (20, 37). Despite increasing evidence about the spontaneous healing and/or the stability of most dissections at follow-up, homogenous guidelines about when to treat DCB-induced acute dissections with bailout stenting are lacking (37).

Kissing balloon inflation after stent implantation, followed by POT, is usually performed in most left main bifurcation lesions treated with a provisional single-stent technique for achieving adequate stent strut opening toward the side branch and to improve jailed side branch area, when residual stenosis is significant. In addition, it is a mandatory step for all two-stent techniques (38). For left main trifurcation lesions, the choice between trissing balloon inflation and sequential double kissing balloon inflation main vessel and each side branch is controversial. The trissing balloon inflation has some theoretical advantages such as the ability to achieve a better-rounded geometry in both stented and non-stented vessels (39, 40). Final POT is considered useful to restore the fractal geometry of the main vessel thus reducing the eccentricity index, ensuring a round LM lumen, and upsizing the stent dimension according to LM diameter (38, 41).

In our case, we used trissing balloon inflation with 3.0 mm semi-compliant balloons, which were easily accommodated in a 7-French guiding catheter, before DCB use. Trissing balloon inflation with larger size non-compliant balloons, such as 3.5 mm, for bigger side branches would have required the use of an 8-French guiding catheter, thus requiring an alternative arterial access, such as the femoral approach in most cases, or alternative techniques such as double radial access with two guiding catheters engaging the left main. We next performed kissing balloon inflation with DCB to both the jailed ostial side branches that were affected by the restenosis, followed by trissing balloon inflation to optimize the geometry of two carinas, and final POT achieving an optimal angiographic result both in the immediate phase and at follow-up.

## Conclusions

Left main trifurcation lesion restenosis represents a challenge for interventional cardiologists. The use of trissing balloon inflation for carina geometry optimization, kissing balloon dilation with DCB without additional stent implantation, and final POT in the main vessel may represent a valuable therapeutic option.

## Data Availability

The original contributions presented in the study are included in the article/Supplementary Material, further inquiries can be directed to the corresponding author.
